# Tau-related white-matter alterations along spatially selective pathways

**DOI:** 10.1016/j.neuroimage.2020.117560

**Published:** 2020-11-12

**Authors:** Qiuting Wen, Shannon L. Risacher, Linhui Xie, Junjie Li, Jaroslaw Harezlak, Martin R. Farlow, Frederick W. Unverzagt, Sujuan Gao, Liana G. Apostolova, Andrew J. Saykin, Yu-Chien Wu

**Affiliations:** a Department of Radiology and Imaging Sciences, Indiana University School of Medicine,, Indianapolis, IN 46202, USA; b Indiana Alzheimer Disease Research Center, Indiana University School of Medicine, Indianapolis, IN, USA; c Department of Electrical and Computer Engineering, Indiana University Purdue University Indianapolis, IN, USA; d University Information Technology Service - Research Technology, Indiana University, Indianapolis, IN, USA; e Department of Epidemiology and Biostatistics, School of Public Health, Indiana University, Bloomington, IN, USA; f Department of Biostatistics, Indiana University School of Medicine, Indianapolis, IN, USA; g Department of Neurology, Indiana University School of Medicine, Indianapolis, IN, USA; h Department of Clinical Psychology, Indiana University School of Medicine, Indianapolis, IN, USA

**Keywords:** Tau PET, Diffusion MRI, WM degeneration, DTI, NODDI

## Abstract

Progressive accumulation of tau neurofibrillary tangles in the brain is a defining pathologic feature of Alzheimer’s disease (AD). Tau pathology exhibits a predictable spatiotemporal spreading pattern, but the underlying mechanisms of this spread are poorly understood. Although AD is conventionally considered a disease of the gray matter, it is also associated with pronounced and progressive deterioration of the white matter (WM). A link between abnormal tau and WM degeneration is suggested by findings from both animal and postmortem studies, but few studies demonstrated their interplay *in vivo*. Recent advances in diffusion magnetic resonance imaging and the availability of tau positron emission tomography (PET) have made it possible to evaluate the association of tau and WM degeneration (tau-WM) *in vivo*. In this study, we explored the spatial pattern of tau-WM associations across the whole brain to evaluate the hypothesis that tau deposition is associated with WM microstructural alterations not only in isolated tracts, but in continuous structural connections in a stereotypic pattern. Sixty-two participants, including 22 cognitively normal subjects, 22 individuals with subjective cognitive decline, and 18 with mild cognitive impairment were included in the study. WM characteristics were inferred by classic diffusion tensor imaging (DTI) and a complementary diffusion compartment model – neurite orientation dispersion and density imaging (NODDI) that provides a proxy for axonal density. A data-driven iterative searching (DDIS) approach, coupled with whole-brain graph theory analyses, was developed to continuously track tau-WM association patterns. Without applying prior knowledge of the tau spread, we observed a distinct spatial pattern that resembled the typical propagation of tau pathology in AD. Such association pattern was not observed between diffusion and amyloid-*β* PET signal. Tau-related WM degeneration is characterized by an increase in the mean diffusivity (with a dominant change in the radial direction) and a decrease in the intra-axonal volume fraction. These findings suggest that cortical tau deposition (as measured in tau PET) is associated with a lower axonal packing density and greater diffusion freedom. In conclusion, our *in vivo* findings using a data-driven method on cross-sectional data underline the important role of WM alterations in the AD pathological cascade with an association pattern similar to the postmortem Braak staging of AD. Future studies will focus on longitudinal analyses to provide *in vivo* evidence of tau pathology spreads along neuroanatomically connected brain areas.

## 1. Introduction

Progressive accumulations of amyloid-*β* and tau protein are hallmark pathologies of Alzheimer’s disease (AD) (Alzheimer et al., 1991; Goedert et al., 1991). While amyloid-*β* forms extracellular senile plaques in the brain gray matter (GM) (Haass and Selkoe, 2007), hyperphosphorylated tau protein forms intracellular neurofibrillary tangles (Bancher et al., 1989). Neurofibrillary tangles often co-occur with amyloid-*β* in the neocortex of the aging brain (Price and Morris 1999; Scholl et al., 2016). Nevertheless, because of differences in microscopic molecular pathways (Bancher et al., 1989; Haass and Selkoe 2007), the anatomic spread of amyloid-*β* and tau follow distinct temporal patterns across AD stages. Unlike amyloid-*β*, which is poorly correlated with clinical symptoms (Nelson et al., 2012), tau pathology is well-correlated with AD severity (Arriagada et al., 1992) and episodic memory decline (Mitchell et al., 2002), and has characteristic anatomic distributions that have been widely adopted for disease staging (Braak and Braak, 1991). Starting from the transentorhinal cortex (stages I and II), tau pathology extends to the adjacent limbic system in the medial and inferior temporal lobes and to the posterior cingulum cortex at stages III and IV (Braak and Braak 1991; Braak et al., 2006; Braak et al., 2011). At later Braak stages (i.e., V and VI), tau pathology spreads to the neocortex of the parietal lobes and frontal lobes (Braak and Braak 1991; Braak et al., 2006; Braak et al., 2011). Additional evidence for this clinicopathologic staging of AD was demonstrated in recent studies using *in vivo* tau positron emission tomography (PET) with [^18^F]Flortaucipir (Scholl et al., 2016; Schwarz et al., 2018).

The intracellular origination of tau protein and its distinct spread pattern suggest that white-matter (WM) deterioration plays a role in AD. One early pathoanatomic study reported changes in WM macro- and microstructures, including demyelination and axonal degeneration, in the brains of patients with AD (Brun and Englund, 1986). Postmortem studies of AD patients have also demonstrated that cortical tau pathology is associated with the WM Wallerian degeneration protease (i.e., calpain) (McAleese et al., 2017) and alterations in WM microstructural integrity detected by diffusion magnetic resonance imaging (dMRI) (Kantarci et al., 2017). A link between tau pathology and WM degeneration is also supported by a recent PET and genetic transcriptome study in which neurogenetic contributions to tau spreading were found to be ‘axon related’ (Sepulcre et al., 2018). Therefore, elucidating the associations between tau and WM degeneration *in vivo* will contribute to a better understanding of their interplay across the AD spectrum and their impact on clinical symptoms.

The development of tau PET tracers and advances in diffusion MRI enable assessment of the relationship between tau deposition in the GM and microstructural alterations in the WM *in vivo*. Recent neuroimaging studies reported detectable associations between tau and WM diffusion alterations in cognitively healthy older individuals, in atypical AD, as well as in individuals with pathologically-staged AD (Kantarci et al., 2017; Jacobs et al., 2018; Strain et al., 2018; Sintini et al., 2019). Particularly, in an antemortem MRI study, alterations in diffusion tensor imaging (DTI) were associated with postmortem Braak neurofibrillary tangle staging (Kantarci et al., 2017). This association pattern involved the crus of the fornix and ventral cingulum tracts. A similar association between DTI and tau deposition detected *in vivo* with tau PET was observed between the anterior temporal cortex and its WM projections in 59 cognitively healthy and 10 cognitively impaired participants (Strain et al., 2018). In a study of 256 cognitively healthy individuals (Jacobs et al., 2018), the spread of tau pathology from the medial temporal lobe to the posterior cingulate cortex of the brain via strategically located structural connections (i.e., hippocampal cingulum bundle) was revealed *in vivo*. Together, the initial evidence suggests an important association between tau and WM degeneration and encourages whole-brain data-driven analyses to validate the extent and scope of such an association pattern across the early AD disease spectrum.

Although both dMRI and tau PET provide whole brain information, previous studies utilizing both imaging modalities have focused on one or a few tracts that were preselected on the basis of prior knowledge. Few studies have taken advantage of the full potential of neuroimaging to investigate brain-wide patterns of associations between tau and WM. In the present study, we explored the overarching hypothesis that the association between tau deposition and WM microstructural alterations (tau-WM association) is present not only in isolated tracts, but in continuous structural connections. To this end, we developed a data-driven iterative searching (DDIS) approach to identify a pattern of associations between tau deposition in GM areas and WM integrity along the connecting tracts. We chose to include a cohort of participants consisting of cognitively normal, subjective cognitive decline, and mild cognitive impairment older adults that are in the early stage of the AD spectrum. Such a cohort likely contains a better gradient of tau deposition with slight, medium to severe tau deposition. Similar approaches were also applied to associations between amyloid-*β* and WM integrity. Metrics for the WM integrity and microstructure were derived from advanced multi-shell diffusion MRI techniques with classic DTI and a complementary diffusion compartment model – neurite orientation dispersion and density imaging (NODDI), that provides an image proxy for axonal density.

## 2. Materials and methods

### 2.1. Participants

Participants from the Indiana Alzheimer Disease Research Center (IADRC) who had tau PET imaging with [^18^F]Flortaucipir, amyloid PET imaging with either [^18^F]Florbetaben or [^18^F]Florbetapir and advanced diffusion MRI data were included in this study. The participants included cognitively normal individuals (CN), as well as individuals with subjective cognitive decline (SCD) and those with mild cognitive impairment (MCI). MCI subjects were identified based on a multidisciplinary clinical consensus panel review aligning with NIH-AA criteria (Albert et al., 2011). Briefly, MCI participants had significant self-reported or informant/clinician-reported complaints about their cognition, as well as a significant deficit (> 1.5 standard deviations below normal) in either memory or another cognitive domain (Albert et al., 2011). SCD participants were identified according to the following criteria: elevated levels of subjective memory concerns reflected by a score of 20 or more on the first 12 items of the 20-item Cognitive Change Index (CCI-20) (Rattanabannakit et al., 2016), with or without increased levels of informant-based concerns (Jessen et al., 2014) and without a measurable cognitive deficit. Older adults without significant memory concerns (12-item CCI total < 20) and without a measurable cognitive deficit were considered CN participants. Exclusion criteria for neuroimaging were significant cerebrovascular disease or malformations; a history of chemotherapy or radiation therapy; current major depression; a history of schizophrenia, bipolar disorder, developmental disability, Parkinson disease, brain surgery, brain infection, or significant head injury (loss of consciousness > 30 min); and/or excessive alcohol consumption. The final cohort included 62 participants: 22 CN, 22 SCD, and 18 MCI. All participants provided written informed consent according to procedures approved by the Institutional Committee for the Protection of Human Subjects at Indiana University School of Medicine.

### 2.2. PET

PET scans were performed using a Siemens Biograph mCT scanner. Emission data were recorded continuously (list mode) over the scan period at relative equilibrium (i.e., after a certain period of uptake time) and rebinned into 5-min frames. Standard PET image reconstruction (ordered-subset expectation maximization) was conducted using the scanner software platform (Siemens; Knoxville, TN, USA) with corrections for scatter and random coincidence events, attenuation (with acquired CT images), and radionuclide decay.

For tau PET, approximately 10mCi of [18F]Flortaucipir (^18^F-AV-1451) was administered intravenously and a 30 min scan was initiated after an uptake time of 75 min. The middle four 5-min frames (80–100 min) were spatially aligned to the subject’s T1-weighted anatomic image, motion-corrected, normalized to the Montreal Neurologic Institute (MNI) space before averaging to create a static image volume, and smoothed with an 8-mm full-width half maximum Gaussian kernel in Statistical Parametric Mapping 8 (SPM8). The smoothed images were intensity normalized to the cerebellar crus to create standardized uptake value ratio (SUVR) images. Median SUVR value was summarized in 84 cortical and subcortical regions-of-interest (ROI) generated by FreeSurfer v6.0 from each subject’s T1-weighted images (Salat et al., 2005) using the Desikan-Killiany atlas (Desikan et al., 2006). Tau positivity was defined as a tau SUVR > 1.23, denoted as “tau+” (Jack et al., 2017; Mishra et al., 2017), and the prevalence of tau+ was defined as the percentage of participants with an SUVR above the threshold in that ROI. Continuous variables for PET images (i.e., no thresholds or cutoffs) were used in the association analyses.

For amyloid PET scans, we used two tracers - [18F]Florbetapir (Amyvid, Eli Lilly and Co., Indianapolis, IN, USA) with a 50-min uptake or [18F]Florbetaben (Neuraceq, Piramal Ltd., Mumbai, India) with a 90-min uptake. Like tau PET, the data were pre-processed using SPM8 and intensity-normalized to the whole cerebellum to create SUVR images. Following the cutoffs values established by Landau et al. (2013), participants with SUVR for [18F]Florbetapir higher than 1.1 and participants with [18F]Florbetaben SUVR higher than 1.2 were considered amyloid-*β* positive. To combine the two PET tracers for the association with diffusion metrics, we converted the SUVR to centiloid unit. The whole cerebellum region of interest was taken from the Centiloid project (http://www.gaain.org/centiloid-project (Klunk et al., 2015)). Both [18F]Florbetapir and [18F]Florbetaben scans were processed with the Centiloid algorithm (a form of data normalization that permits grouping data from different amyloid tracers) at the voxel level (Klunk et al., 2015; Risacher et al., 2017). Similar to tau PET, the median [18F]Florbetapir and [18F]Florbetaben uptake values (centiloid unit) were summarized for each cortical and subcortical ROI.

### 2.3. MRI

MRI data were acquired on a single Siemens Prisma 3T scanner with a 64-channel RF receiver head coil. All participants underwent T1-weighted imaging and multi-shell diffusion MRI. T1-weighted anatomical imaging used a 3-dimensional magnetization rapid gradient echo (MPRAGE) sequence with imaging parameters matching the Alzheimer’s Disease Neuroimaging Initiative 2 protocols (http://adni.loni.usc.edu/methods/documents/mri-protocols/). The diffusion MRI protocol employed a single-shot spin-echo echo-planar imaging sequence with a hybrid diffusion imaging (HYDI)-encoding scheme that contained three zero diffusion-weighting (i.e., *b*-value=0 s/mm^2^) and five concentric diffusion-weighting shells (*b*-values = 250, 1000, 2000, 3250, and 5000 s/mm^2^) for a total of 142 diffusion-weighting gradient directions (Wu and Alexander, 2007; Wen et al., 2019). The field of view was 240 × 240 mm with an imaging matrix of 120 × 120 and 68 slices with a slice thickness of 2 mm, yielding 2-mm isotropic voxels. An additional *b* = 0 s/mm^2^ with reversed-phase encoding was acquired for geometric distortion correction. The T1-weighted images were used to generate ROIs for summarizing tau and amyloid deposits in the GM (described above in the PET section).

Diffusion MRI data were first pre-processed using previously described pipelines (Kodiweera et al., 2016; Wu et al., 2018; Wen et al., 2019) for noise reduction (Manjon et al., 2013), motion and distortion correction (FSL *topup* and *eddy* commands). The pre-processed diffusion data were then used to compute diffusion metrics of DTI and NODDI. The DTI metrics were computed on the first two shells (*b*-value = 250s/mm^2^ and 1000s/mm^2^), including fractional anisotropy (FA, the coherence of microstructure water diffusion), mean diffusivity (MD, the magnitude of overall water diffusion), and supplementary analyses of axial (Da, along the principal water diffusion direction (Kim et al., 2006)) and radial diffusivity (Dr, perpendicular to the principal water diffusion direction (Song et al., 2005)) (FSL *dtifit* command). All five shells were used in the NODDI analysis with the AMICO toolbox (https://github.com/daducci/AMICO (Daducci et al., 2015)). The NODDI approach provides complementary diffusion metrics that may be biologically more specific than DTI by modeling the water diffusion signal according to one of three different pools (Zhang et al., 2012): (i) free water diffusion (such as in cerebrospinal fluid); (ii) intracellular restricted diffusion inside dendrites and axons, and (iii) extracellular hindered diffusion. The resultant indices included in the analysis are the intracellular volume fraction (ICVF), a proxy of axonal density, and orientation dispersion (OD), a measure of the degree of fanning in axonal orientations (Kodiweera et al., 2016). Supplementary Table S1 summarizes the diffusion metric acronyms and their microstructure implications. The six diffusion maps were then used to extract tract-specific values after streamline tractography.

**Fig. 1.**
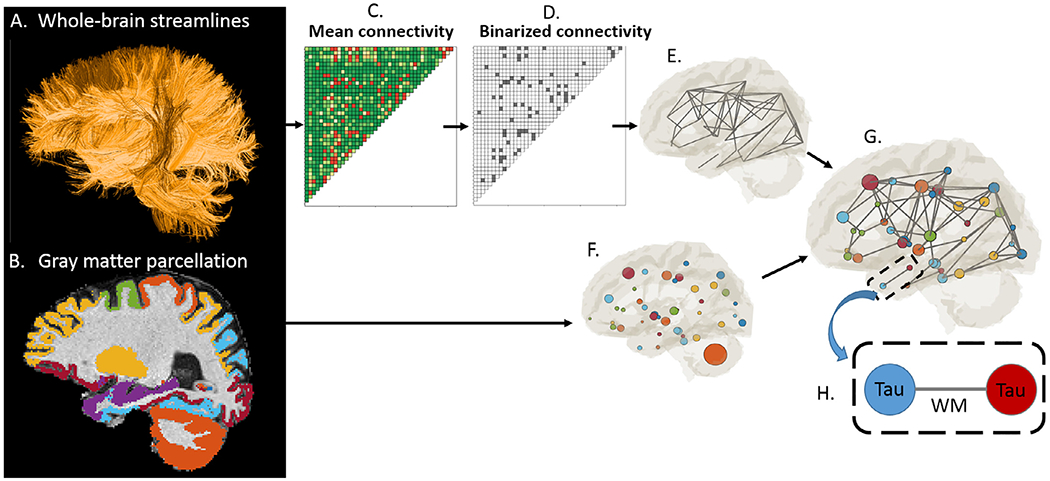
Establishing the backbone connection. The whole-brain streamlines (A) generated from tractography were classified according to their end-points (i.e., 84 cortical/subcortical ROIs (B)) to produce a structural connectivity matrix, where each element indicates the number of streamlines connecting any pair of ROIs. To eliminate false-positive connections arising from imaging noise, the mean connectivity matrix of all subjects (C) was binarized with a threshold of 10% (D). For each subject, tract-specific diffusion metrics were derived along all binarized connections, and tau-PET signals were summarized in all ROIs (H). Thus, “tau-WM” could be analyzed on “node-edge” pairs. Circle size in the brain illustration indicates the ROI size with a matching color code in (B, F, and G).

WM streamline tractography was performed using all 5-shell diffusion data with MRtrix3 guidelines (https://mrtrix.readthedocs.io/en/latest/quantitative_structural_connectivity/ismrm_hcp_tutorial.html (Tournier et al., 2012)). In brief, subject whole-brain streamlines were generated using the multi-shell, multi-tissue constrained spherical deconvolution and probabilistic tracking algorithm (maximum tract length = 250 mm, FA cutoff = 0.06). A structural connectivity matrix between 84 GM ROIs was generated, where each element indicates the number of streamlines connecting any pair of ROIs, or the strength of the connection between the two ROIs. Instead of performing the analysis on all connections, which would include noise connections, we restricted the analysis to a “backbone” connection structure where only strong connections that are common to all subjects were preserved. Fig. 1 illustrates the workflow for backbone generation. The subject-level connectivity matrixes were normalized by each subject’s whole-brain streamline counts and corrected for ROI sizes to remove variations arising from individual brain or ROI sizes. Afterwards, all subjects’ connectivity matrixes were averaged (Fig. 1C) and binarized with a threshold of 10% (of maximum streamline counts) (Fig. 1D). The final binarized connectivity (Fig. 1D) described a backbone connection structure (i.e., edges, Fig. 1E) used for the analysis that is common to all subjects. The per subject tract-specific diffusion measures (i.e. FA, MD, Da, Dr, ICVF, OD) were extracted on each connection of the backbone. The tau-PET signal in the GM ROIs (Fig. 1F) and diffusion metrics along the WM edges (Fig. 1E) were used in the pathway searching, where the “tau-WM” associations can be analyzed on the “node-edge” segments (Fig. 1H).

### 2.4. Data-driven iterative searching approach (DDIS)

**Fig. 2.**
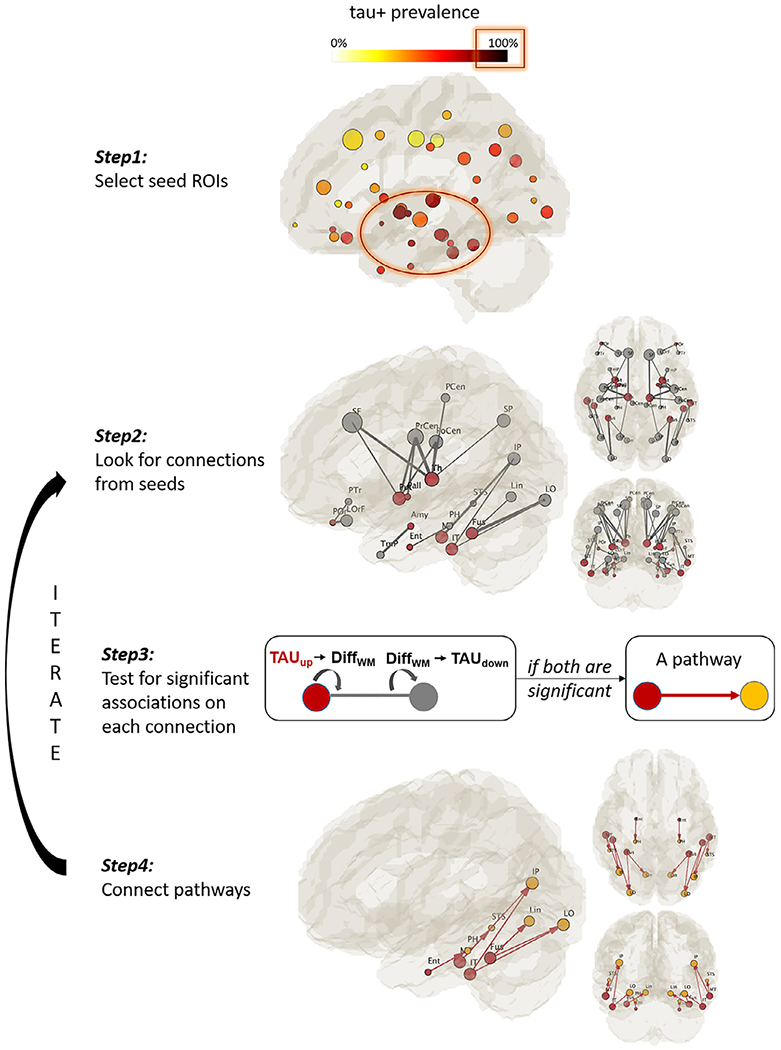
Pathway searching pipeline. Step1 selects seed ROIs with high tau-PET signals among the subjects (i.e., tau+ prevalence > 80%). Step2 identifies all possible connections from the seeds (gray sticks). For each connection, two linear regressions were performed as shown in Step3. If both tests revealed significant results, the connection was considered an association pathway and was highlighted in Step4. Steps 2–4 were reiterated until no further pathways were detected.

DDIS was developed to search for tau-WM associations in continuous structural connections. The search started from seed ROIs with the highest tau+ prevalence (i.e., > 80%, defined as the percentage of subjects having tau+ in a given ROI, Fig. 2, Step1). Afterwards, all possible structural connections from the seeds were identified on the backbone (Fig. 2, Step2 gray sticks). For each connection, we applied two generalized liner regression models testing the following two hypotheses (Fig. 2 Step3):

1. if the tau-PET signal in a GM seed (TAU_up_: explanatory variable) is associated with a diffusion metric along its connecting WM tract (Diff_WM_: dependent variable); and
2. if the same diffusion metric along the WM tract (Diff_WM_: explanatory variable) is associated with tau-PET signal in the tract’s destination GM ROI (TAU_down_: dependent variable).

**Fig. 3.**
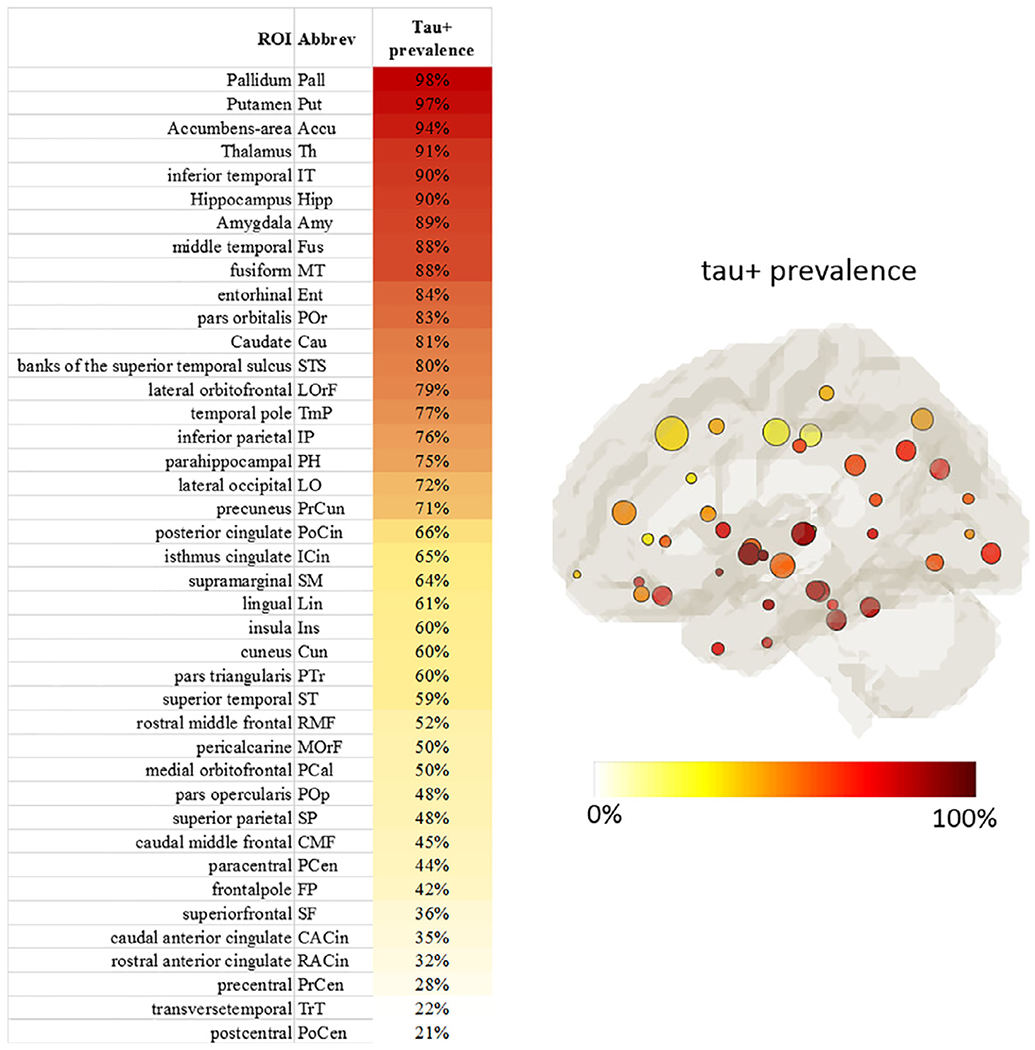
Tau+ prevalence of all ROIs. Left: ROIs sorted according to the Tau+ prevalence. Right: The map of ROIs color-coded by the tau+ prevalence level with circle size denoting the ROI size. Left and right hemispheres demonstrated very similar patterns and were averaged.

An association pathway was deemed to exist if both regressions were significant (*P* < 0.05) (Fig. 2, Step4). The destination ROIs (Fig. 2, yellow ROIs) of the association pathways then became new seeds and the search continued by iterating Step2 to Step4 until no further pathways could be detected. The left and right hemisphere association patterns were analyzed independently. In addition, to avoid circulations within seeds during the search, connections among the seed ROIs were not considered. The directionality of Fig. 3 Step3 is necessary for the appropriate interpretation of the two hypotheses with the covariates (sex and age) on the right side of the general linear regression equation. This is also the building blocks of the iterative approach in DDIS.

The diffusion data were used for two purposes. Firstly, it was used to perform WM streamline tractography to determine the connections between GM ROIs. Secondly, it was used to derive diffusion metrics that can probe microstructure properties along the WM connections. More specifically, the diffusion metrics were extracted from WM edges, not nodes. The nodes were generated with GM parcellation, and only tau deposition was quantified in the nodes. The connections (i.e. edges) between the nodes were determined with WM tractography. The integrity of the WM connection was interrogated by the diffusion metrics (i.e., the DTI metrics: FA, MD, Da, and Dr; and the NODDI metrics: ICVF and ODI). The same connectivity matrix (i.e., backbone connections or edges) was used for all the diffusion metrics. As DDIS applies linear regression between tau in the nodes and diffusion metric along the edges, DDIS produces an association pattern that is specific to a diffusion metric. For example, by saying “apply DDIS to MD”, it means MD is used in the regression to probe the WM integrity in the edges.

To test the stability and reproducibility of the DDIS framework, we performed additional analyses on the choice of thresholds and used a bootstrap method to assess the reproducibility and reliability. There are only two predetermined thresholds required in the DDIS approach (i.e., the threshold in creating binary backbone connection and the threshold in the seed selection). The purpose of the threshold in creating backbone connection is to minimize the false positive connection arising from the image noise in diffusion MRI while preserving the majority of the valid connections. To this end, we used a liberal threshold of 10% (of maximum streamline counts). Thresholding in creating binarized connection (i.e., edges) has been previously discussed and used in structural connectivity (Rubinov and Sporns, 2010; Mijalkov et al., 2017). Here, we demonstrated the distribution of streamline counts and assessed the results of the association pattern at difference thresholds with a 1% increment from 4% to 11% (Supplementary Fig. S1). The seed selection was performed by thresholding the prevalence of the tau positivity among the study population. The choice of the seed-selection threshold is to balance between the ratio of the seeds to the total number of nodes and the ratio of off-target nodes to the seeds. The effect of the number of initial seeds on the association pattern was evaluated (Supplementary Fig. S3). In addition, the number of seeds changes slightly with a different population composition. Such effect on the stability of the DDIS approach was evaluated in the bootstrap procedure described below.

To test the reproducibility of pathway searching and avoid type 1 error, bootstrap resampling with replacement was performed on the population, with fixed sample size (62 participants) in each bootstrap. For each bootstrap sample, the whole pipeline in Fig. 2 was repeated, including calculation of tau+ prevalence and seed selection. The discovered pathways of all bootstrap samples were combined and summarized through a voting system. Note that the bootstrap procedure is not part of the DDIS approach, but serves as a sanity check in this study to test the reproducibility and reliability of the DDIS approach. For a fixed population (i.e., sample), there is no randomness once the seed ROIs are selected. For a modest sample size, the observed pattern may be biased by the type 1 error. Therefore, the purpose of the bootstrapping in this study was to evaluate the stability of the association pattern when the population was randomly resampled.

### 2.5. Statistical analyses

In DDIS, regression analyses were controlled for possible confounders: age and sex. For demographic and cognitive variable comparisons, ANOVAs were employed with Tukey’s post-hoc or χ^2^ tests for categorical variables. A threshold of *P* < 0.*05* was considered significant for all statistical models. Statistical analyses were conducted using R-3.5.1.

## 3. Results

### 3.1. Subject characteristics

Demographics and neuropsychological comparisons between the groups are shown in Table 1. No differences in age, sex, or education were detected between groups. Compared with the CN group, the SCD group had a higher self-reported Cognitive Change Index (CCI-self, 12 items) by design, but did not differ in other neuropsychological test scores. Individuals with MCI had significantly lower scores on the Rey Auditory Verbal Learning Test (RAVLT) immediate recall and delayed recall, and on the Montreal Cognitive Assessment (MoCA), as well as a significantly higher Clinical Dementia Rating scale (CDR) – sum of boxes and CCI.

### 3.2. The prevalence of tau positivity

The tau+ prevalence across all subjects for each ROI is summarized in Fig. 3 with high tau PET SUVR localizing in the basal ganglia and medial temporal regions. Supplementary Fig. S2 lists the tau+ prevalence in each group. While all groups had a similar pattern of tau gradients across ROIs, the MCI group had a higher tau+ prevalence than the CN and SCD groups in almost every ROI. The ROIs with tau+ prevalence greater than 80% among all subjects were used as seeds for initiating the pathway searching process. The rationale for the threshold selection is discussed below (in Identified pathways session).

### 3.3. Identified pathways

The DDIS analysis was performed on all the diffusion metrics described above in Materials and Methods (also in Supplementary Table S1). Due to the similarity in the results, here we focus the description of the association patterns on DTI-derived mean diffusivity (MD). Nevertheless, results of all diffusion metrics are summarized below in the Diffusion metrics section.

**Fig. 4.**
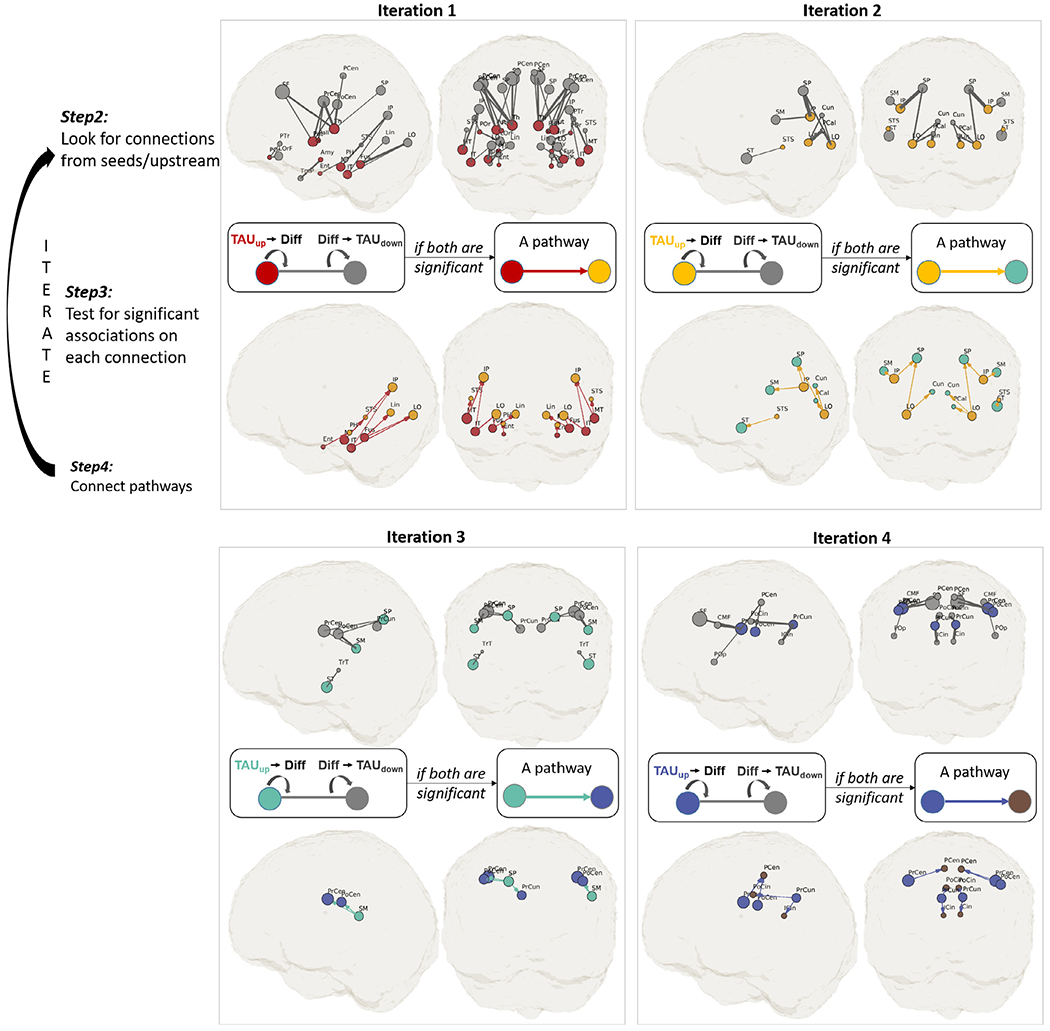
An illustration of the iterative approach in DDIS. The searching started from initial 14 seed ROIs (red circles in Iteration 1) clustered mainly in the medial temporal lobe. All possible connections from the seed ROIs were initially colored gray (top row). Two linear regressions were performed on each connection (middle row) to determine TAU_up_-MD-TAU_down_ associations. The significant pathways were assigned colors (bottom row). The colored ROIs (TAU_down_) served as seed ROIs (TAU_up_) in the next iteration. Note that the purpose of this figure is to illustrate the behind-the-scene process of the iterative approach. The identified/survived pathways and nodes were listed in Fig. 5 with full names and matched colors. The full form of the abbreviations is also listed in Fig. 3 left two columns.

Fig. 4 shows the detected pathways at each iteration. The searching analysis started from the seed ROIs (Fig. 4, red circles, tau+ prevalence > 80%) located in the basal ganglia and medial temporal lobe. The threshold for seed ROIs (i.e., 80% tau+ prevalence) is arbitrary. Similar results were observed for other seed thresholds explored in Supplementary Fig. S3. Although the seeds had WM tracts connecting to many brain areas (gray sticks and circles in Fig. 4, Iteration 1 top panel), only posterior pathways showed significant tau associations with WM integrity detected by MD (Fig. 4, Iteration 1 bottom). The association pathways identified in each iteration were assigned a color (bottom row). The colored destination ROIs (TAU_down_) served as seed ROIs (TAU_up_) in the next iteration. Overall, the association pattern developed posteriorly, then superiorly, and eventually terminated anteriorly in the postcentral region. The searching continued for four iterations. In the fifth iteration (not shown), none of the connections from the brown-seeded ROIs showed significant associations in the downstream connections (*P* > 0.05).

**Fig. 5.**
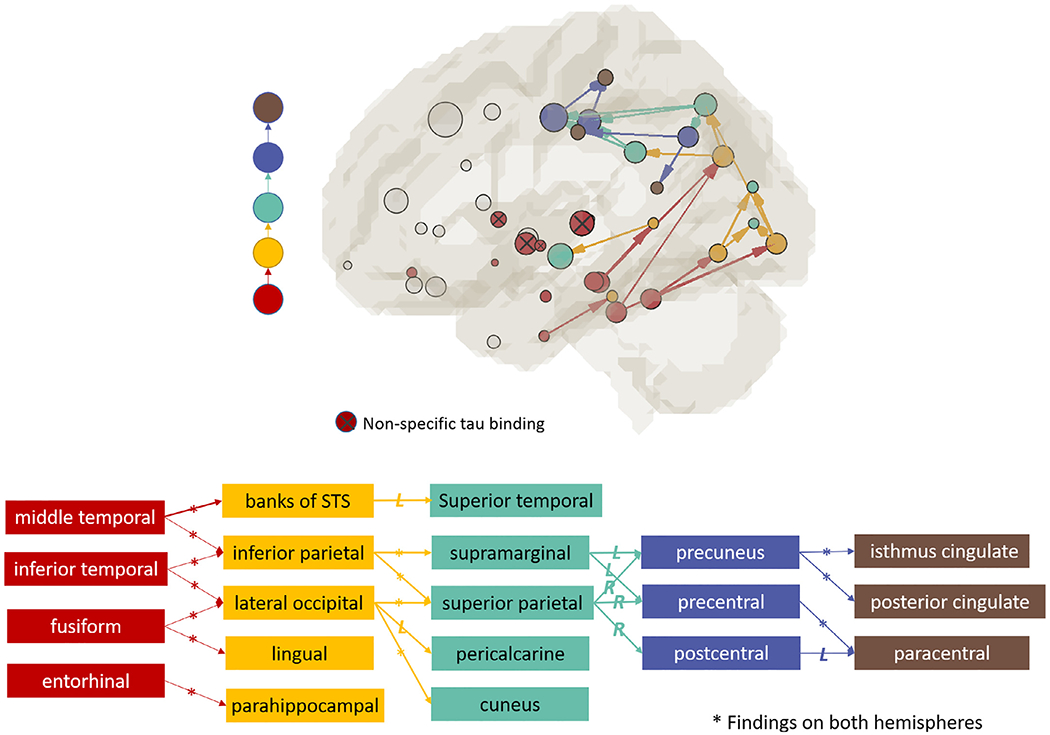
Summary of identified pathways for the tau-PET signal and MD association. Each colored pathway contains a dual association between tau-PET signal at both ends and MD in the tract. Involved GM ROIs are listed below with matching colors representing the stages/iterations in DDIS in Fig. 4. Out of 22 identified pathways, 14 were found in both hemispheres, indicated by a star (*) on the connections in the bottom figure. The red circles with a cross inside the circle, ⊗, denote those initial seed ROIs known to have off-target tau bindings. These ROIs were located in the basal ganglia, including the pallidum, caudate, putamen, and thalamus. STS: superior temporal sulcus. L: left hemisphere. R: right hemisphere.

Fig. 5 summarizes all the identified pathways for the tau-MD associations. Out of the 22 pathways, 14 were found in both hemispheres, indicated by a star (*). Higher tau levels were associated with increased MD in all pathways (*P* < 0.05). Some of the initial seed ROIs are known to have off-target tau binding (i.e., pallidum, caudate, putamen, thalamus, indicated by the red ⊗ in Fig. 4). These ROIs were treated the same way as the rest ROIs in the analysis. They consistently showed no significant pathways across the bootstrapping validation. Among the identified pathways (*P* < 0.05), quite a few associations had *P* values smaller than 0.01 (Table 2). Most of the earlier pathways that connect medial temporal lobe to the posterior part of the brain had lower *P* values (i.e., *P* < 0.001, blue). For example, the *P* value for the connection between inferior temporal and inferior parietal pathway had a *P* value of 0.00003 for the TAU_up_-Diff_WM_ association and 0.00006 for the Diff_WM_ - TAU_down_ association.

### 3.4. Bootstrapping

**Fig. 6.**
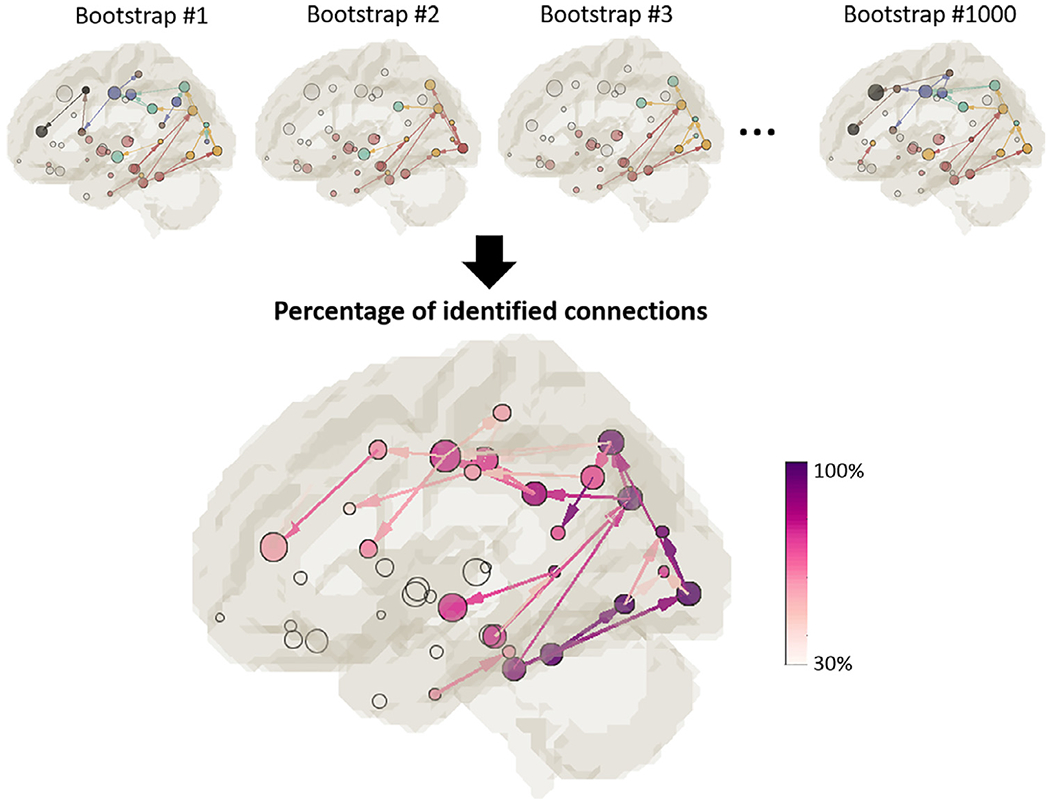
Bootstrapping results. Top row: Detected pathways for each bootstrap sample. Bottom row: The map summarizing the 1000 bootstrap results. The color scale denotes the percentage of bootstrap samples for which the TAU_up_-MD-TAU_down_ pathways were identified. Pathways with fewer than 30% are not shown.

Bootstrap sampling was performed 1000 times and the results are summarized in Fig. 6 for the tau-MD association. In each bootstrap sample, the subjects were randomly selected with replacement from the original pool, and thus the initial seed ROIs differed as well. While the detected pathways for each bootstrap sample (Fig. 6, top row) differed as expected, they followed a similar pattern that initiated from the medial temporal regions, posteriorly to occipital regions, and superiorly-anteriorly to the parietal or frontal regions. This pattern can be better appreciated in the summary map at the bottom of Fig. 6, where some persistent pathways appeared more than 90% of the time, including inferior temporal → inferior parietal → superior parietal, inferior temporal → lateral occipital → cuneus, and fusiform → lingual. To demonstrate the level of confidence for each established pathway in the main pattern (Fig. 5), the percentage of connections identified in bootstrap samples were summarized in Supplementary Fig. S4.

### 3.5. Diffusion metrics

**Fig. 7.**
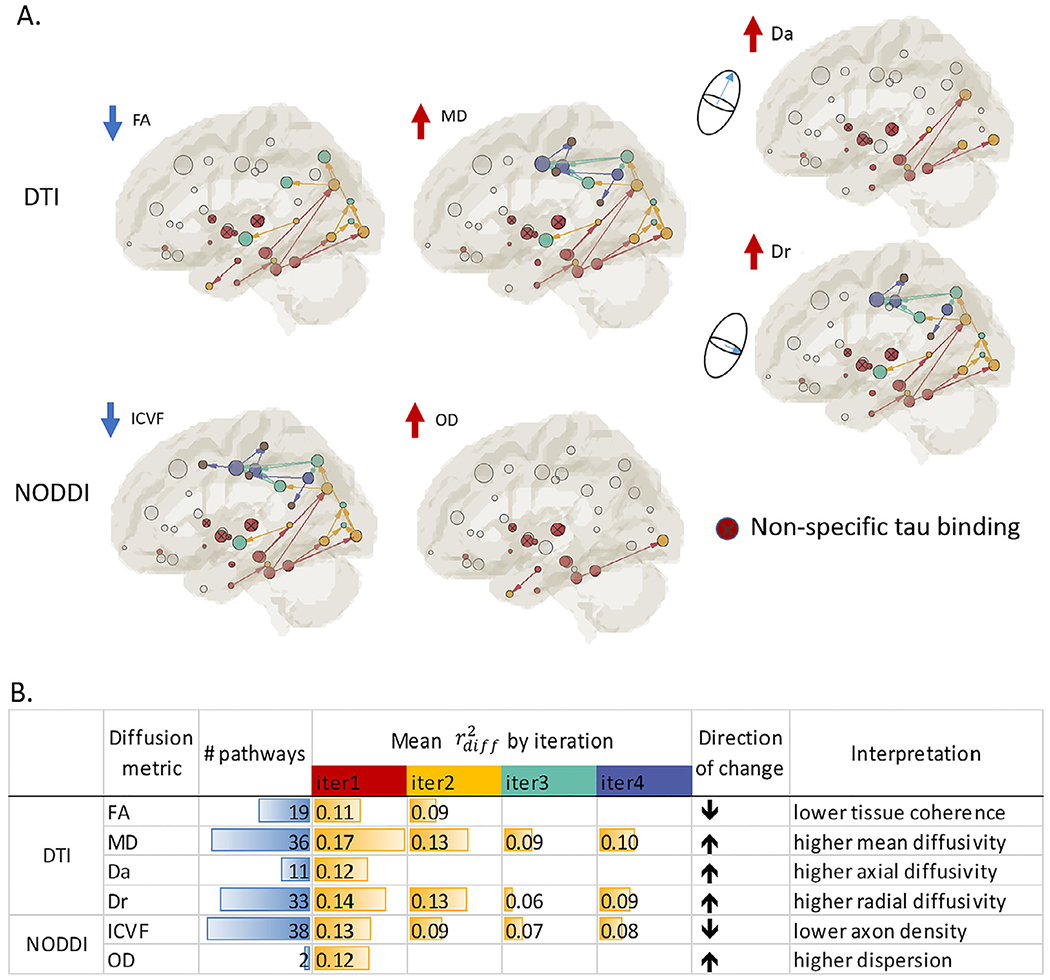
Summary of identified patterns (A) and statistics (B) for all the diffusion metrics. A. In the DTI-derived metrics, higher tau-PET signal is consistently associated with decreased FA and increased MD, Da and Dr. In the NODDI-derived metrics, higher tau-PET signal is associated with decreased intracellular volume fraction (ICVF) (i.e. a proxy for axonal density). Increased OD was detected in very few (i.e. 2) pathways. Independent DDIS on Da and ICVF revealed similar patterns to that of MD. B. Statistics of the association patterns. “# pathways” denotes the total number of detected pathways, each of which contains a dual association between tau in both ends and a diffusion metric in the connection (i.e. color-coded connections in A). Adjusted r^2^_*diff*_ describes the additional variance of tau-PET signal explained by a diffusion metric in the multivariate regression model that controls for age and sex in the detected pathways. Only significant r^2^_*diff*_ (i.e., *p* < 0.05) were listed here. “Direction of change” denotes the direction of change in the diffusion metrics when tau-PET signal increases in the adjacent GM ROIs.

DDIS was applied to six diffusion metrics respectively and the association patterns are summarized in Fig. 7. For DTI-derived metrics, higher tau-PET signal is consistently associated with decreased FA and increased MD. Comparing the two components of MD, increased MD is driven by increased diffusivity along both radial (Dr) and axial (Da) directions in the early iterations (i.e. red-yellow), and is driven by increased diffusivity along the radial direction in the later iterations (i.e. yellow-green-purple-brown). For the NODDI metrics, independent DDIS on ICVF revealed a similar spatial pattern to that of MD, but with decreased ICVF (i.e., a proxy for axonal density). Increased OD was only detected in a few (i.e. 2) pathways. Fig. 7B summarizes the total number of detected pathways for each diffusion metric and corresponding *r^2^_diff_* (describing the additional variance of tau-PET signal explained by a diffusion metric in the linear regression) by iterations. As expected, *r^2^_diff_* are highest in the earliest iteration and becomes lower in the later iterations.

### 3.6. Amyloid-diffusion (amy-diff) association patterns

As a supplementary analysis, DDIS framework was performed on amyloid-*β* PET data to investigate the amy-diff association patterns by replacing tau-PET signal with amyloid PET signal in the linear regression models. Seeds were selected based on the amyloid-*β* prevalence map (Supplementary Fig. S2) that includes precentral, pericalcarine, paracentral, lateralorbitofrontal, banks of the superior temporal sulcus, Pallidum, Putamen and Thalamus. None of the diffusion metrics were found to have major association patterns with amyloid-*β* (i.e., number of detected pathways ≤ 5, Supplementary Fig. 5).

## 4. Discussion

The present study investigated the relationship between GM tau deposition and WM degeneration using a whole-brain data-driven method on a cross-sectional sample. We found a high degree of association between GM tau-PET signal and WM integrity probed by both DTI and NODDI metrics. The distinct spatial association pattern initiated from the medial temporal lobe and developed posteriorly to the occipital brain regions and superiorly anteriorly to the parietal and frontal lobes, similar to what is described by Braak staging, a postmortem neuropathological staging method for AD (Braak and Braak 1991; Braak et al., 2011; Scholl et al., 2016). Such similarity suggests that WM may play an important role in facilitating the deposition of tau pathology. Our study also provides *in vivo* evidence that WM degeneration, as have been previously observed in patients with cognitive decline (McAleese et al., 2018; Mito et al. 2018a, b; Wen et al., 2019), could be tau related.

Our data-driven results support previous longitudinal studies, albeit with limited anatomical scope, that WM vulnerability facilitates tau propagation in AD. In a study of autosomal-dominant AD, the posterior parietal region was among the earliest affected regions showing degeneration approximately 5 to 10 years before the onset of clinical symptoms (Caballero et al., 2018). In a healthy older population, hippocampal cingulum bundle diffusivity predicted tau accumulation in the downstream-connected posterior cingulate cortex in the at-risk (i.e., amyloid-positive) subjects (Jacobs et al., 2018). Together, these previous findings and our results suggest that WM alterations are an intrinsic part of the AD pathophysiological cascade. Further validating WM diffusion metrics as a clinically relevant biomarker to monitor or even predict tau propagation may be achieved via longitudinal studies with the proposed whole-brain data-driven method.

The multi-shell HYDI diffusion acquisition enabled compartment model fitting to extract biologically relevant diffusion metrics that may provide more specific interpretation compared to DTI. In this study, the NODDI model provided complementary diffusion metrics that may clarify the underlying mechanisms of WM microstructural changes in association with the increased tau-PET signal. Our results demonstrated that tau-related WM degeneration was best characterized by increased mean and radial water diffusivity as probed by DTI-derived MD and Dr, and decreased axonal density as probed by NODDI-derived ICVF. The overall trend of these diffusion metrics suggested a lower axonal packing density accompanied by a high degree of radial diffusion freedom. The decrease in ICVF but lack of findings in OD suggested that the orientation of the degenerated WM tracts was unaffected in spite of decreasing density. Potential reasons for the tau-related WM alterations could be (1) hyperphosphorylation of tau decreases binding between microtubules, causing deterioration of intra-axonal cytoskeleton integrity (Alonso et al., 1996), and/or (2) Wallerian degeneration of axons and demyelination as a consequence of the increased cortical burden of tau pathology, as observed in a postmortem study (McAleese et al., 2017). Interestingly, the positive correlations of tau and Da were mainly concentrated along the pathways of early iterations. The concentrated association pattern indicated that these early pathways connecting the medial temporal lobe and parahippocampal or inferior parietal lobe sustained more extended WM deterioration in both radial and axial directions.

**Fig. 8.**
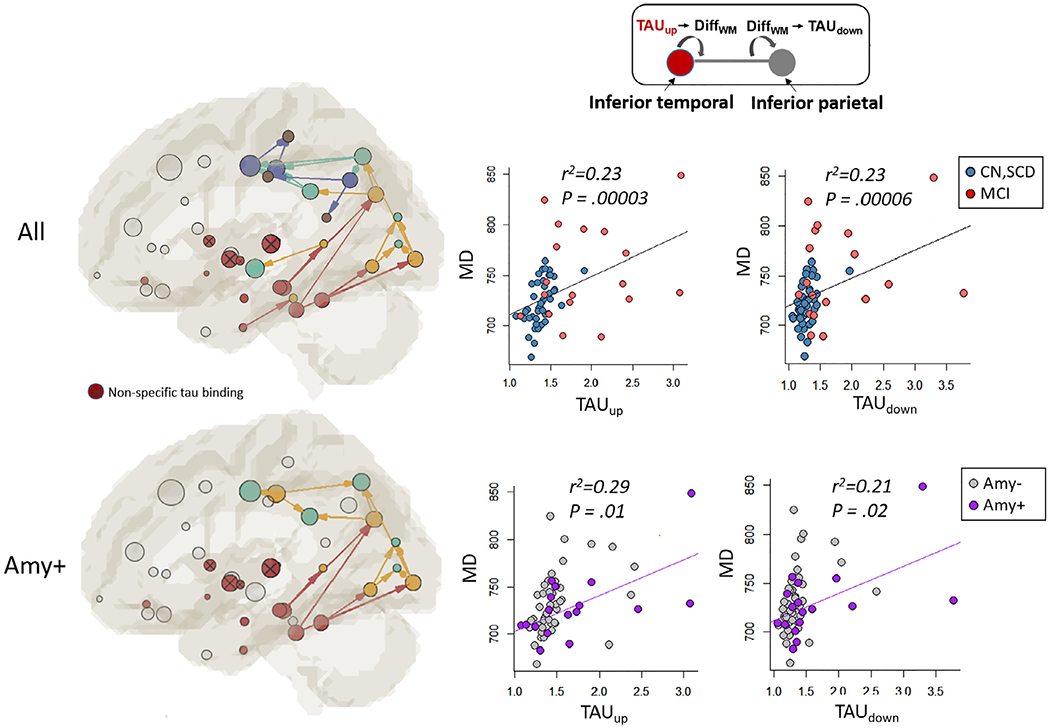
The tau-WM association patterns between the full sample and amyloid positive participants. Top row left: the association pattern derived from all the participants. Top middle: the scatter plot for the tau-PET signal in the seed GM (TAU_up_) vs. mean diffusivity (MD) along the connecting WM tract in one of the early pathways (i.e., the inferior temporal to the inferior parietal ROI). Top right: in the same pathway, the scatter plot for MD along the WM tract vs. the tau-PET signal in the destination GM ROI (TAU_down_). The black regression line represents all the participants including cognitively normal (CN, blue dots), individuals with subjective cognitive decline (SCD, blue dots), and with mild cognitive impairment (MCI, red dots). The r^2^ values are the adjusted r^2^ (coefficient of determination) describing additional variance explained by the independent variable in addition to the covariates (i.e., age and sex). *P* denotes the *P* value of the linear regression. Bottom row left: the association pattern derived from the amyloid positive participants (*n* = 17, Table 1). Bottom middle: the scatter plot for TAU_up_ vs. MD in the same pathway (inferior temporal to the inferior parietal) in the amyloid positive participants (purple dots). Bottom right: the scatter plot for MD vs. TAU_down_. The purple regression line represents the amyloid positive participants only.

Similar tau and WM degeneration association pattern was observed in participants with positive amyloid PET signals, albeit with a small sample size (*n* = 17, Table 1). Compared to the full sample size (Fig. 8, top row), the tau-WM association pattern in the amyloid positive participants demonstrated similar pathways from medial temporal → occipital → parietal → paracentral regions (Fig. 8, bottom row). In the amyloid positive participants, the earlier pathways (i.e., iterations 1-red, 2-yellow, and 3-green) were preserved, whereas the later pathways (i.e., iterations 4-purple and 5-brown) were undetectable. The undetectable, insignificant associations may be caused by the small sample size, which can be observed in the scatter plots (Fig. 8, right panels). Despite with similar adjusted r^2^ values, the *P* values from all the participants (both red and blue dots) were much smaller (*P* = 0.00003 and 0.00006) than the amyloid positive participants (purple dots) (*P* = 0.01 and 0.02). Nevertheless, it is encouraging to observe similar results in the amyloid positive brain with such a small sample size. Future work will continue to focus on the interaction effect of amyloid positivity to the tau and WM degeneration pattern when a large sample becomes available.

Two negative control results were observed supporting the reliability of the positive findings. First, the tau-PET signal in GM ROIs with known off-target binding (Fig. 5) are not expected to have association with WM degenerations, as the PET signal with the ^18^F-flortaucipir tracer in these ROIs are related to iron load instead of tau tangles (Marquie et al., 2015). Nevertheless, we included these ROIs in the analyses to serve as negative controls. The negative findings in these ROIs supported our hypotheses. Since these off-target ROIs did not have any diffusion correlations, they did not contribute to the tau-diffusion association patterns. Thus, the results were the same with and without these ROIs. Second, in the supplementary analysis, similar searching analyses on amyloid-*β* did not identify strong association pattern with very few detectable pathways (≤ 5 as compared to tau ≤ 38) across the diffusion metrics. These findings support previous negative findings on the association between amyloid-*β* and WM degeneration in postmortem studies (McAleese et al., 2015, 2017) and between amyloid-*β* and DTI in an antemortem neuropathological study (Kantarci et al., 2017). A network analysis showed that tau, but not amyloid-*β*, was associated with WM integrity loss and multiple cognitive functions (Pereira et al., 2019). The lack of associations between amyloid-*β* and WM degeneration could be attributed to its genetic origins. A recent neurogenic study identified distinctive pathways for tau and amyloid-*β* accumulation, i.e., the tau-specific genetic profile was classified as ‘axon-related’ and the amyloid-*β* profile as ‘dendrite-related’ (Sepulcre et al., 2018). While not directly associated with WM integrity, a more complex interplay between amyloid-*β* and tau and their effects on WM degeneration could exist, such that amyloid-*β* accumulation may potentiate the propagation of tau (Khan et al., 2014; Pooler et al., 2015; Jacobs et al., 2018). Due to a small number of amyloid-positive subjects (17 of 58), this study is underpowered to infer the interaction effects of amyloid-*β* on the association pattern. Nevertheless, it may be interesting to investigate if the observed tau-WM association patterns may be enhanced with the presence of amyloid-*β* in future studies.

The emphasis of this study is to investigate the role of WM integrity in tau deposition, driven by the hypothesis that tau deposition in the GM ROIs is associated with WM integrity along their connections. With multi-shell diffusion MRI and higher-order streamline tractography model, we were able to resolve crossing fibers and establish cortex-to-cortex connections for any pair of ROIs. We performed the quantification directly along the tractography streamlines in the native scan space without the need for warping template tract ROIs to the subject space as performed in other tract-based studies (Jacobs et al., 2018; Strain et al., 2018). This approach overcame the partial volume issue and guaranteed the correspondence between tract and projected ROIs, as well as the subject specificity of the tract profile. Building upon the WM connectivity backbone, we demonstrated that the WM-tau association pattern resembles previously reported tau propagation patterns. Nevertheless, while WM degeneration may play an important role in tau deposition, the underlying mechanism of tau propagation could be multifactorial. Such complexity may partially explain why some of the GM ROIs (e.g., medial orbitofrontal cortex), despite demonstrating early tau deposition in the postmortem and tau-PET studies (Braak and Braak 1991; Braak et al., 2011; Scholl et al., 2016), did not appear in the WM-tau association patterns.

This study may be limited by the sample’s characteristics. While the pathway search approach is data driven, the results could be sample-dependent and susceptible to the sample size. To test the robustness of our findings, we performed bootstrap sampling and observed high consistency in the identified pathways. In addition, similar pathway patterns (either long or short) were detected across the diffusion metrics, indicating high reliability and reproducibility of the approach. Because of the small sample sizes preventing within group association analyses, global disease severity may contribute to the tau-WM associations. Nevertheless, the pathomechanistic relationships between tau and diffusion changes still play a significant role with *P* < 0.001 after adjusting for group memberships in the inferior temporal → inferior parietal pathway in a retrospective test (results not shown).

The seed selections have been rigorously investigated in this study with bootstrapping and with varying tau+ prevalence thresholds (Supplementary Fig. S3). To account for different distributions of tau-PET signals in different brain regions, alternative approaches may combine fine-tuned tau+ thresholds using z transformation in different GM ROIs (Cho et al., 2016; Vemuri et al., 2017). In addition, to increase the sensitivity of the tau-PET signal to neurofibrillary tangles (aggregates of hyperphosphorylated tau protein) in GM and increase the dynamic range of the association analyses, partial volume correction may be considered in future studies with the Rousset geometric transfer matrix method (Rousset et al., 1998; Baker et al., 2017; Maass et al., 2017).

## 5. Conclusion

This study demonstrated that WM degeneration is associated with the deposition of tau pathology in spatially selective pathways. A lower axonal packing density accompanied by a high degree of radial diffusion freedom along the WM connections were associated with high tau deposition in their projecting GM regions. Such tau-WM associations demonstrated a unique pattern that resembles tau propagation across AD stages. Future longitudinal studies with adequate sample sizes on amyloid-*β*+ populations may provide additional in-vivo evidence of WM-facilitated temporal propagation of tau pathology in preclinical AD.

## Supplementary Material

Supplemental Material

## Figures and Tables

**Table 1:** Demographics and neuropsychological performance.

	CN (*n* = 22)	SCD (*n* = 22)	MCI (*n* = 18)	*P* value	Post-hoc (*P* < 0.*05*)	*N*
Age (years)	69.1 (7.7)	68.9 (7.6)	71.3 (9.3)	ns	ns	22/22/18
Sex (male, female)	4, 18	8, 14	8, 10	ns	ns	22/22/18
Education (years)	17 (2.3)	17.1 (2.7)	16.4 (2.8)	ns	ns	21/22/18
Tau PET positive (−, +) ^†^	17, 5	18, 4	6, 12	n/a	n/a	22/22/18
A*β* PET positive (−, +) ^‡^	20, 2	15, 7	6, 8	n/a	n/a	22/22/14
APOE *ε*4 positive (−, +)	18, 4	16, 6	7, 11	n/a	n/a	22/22/18
RAVLT - immediate recall	46.7 (9.4)	44.5 (7)	30.5 (7.4)	< 0.001	CN,SCD > MCI	18/20/17
RAVLT - delayed recall	10.2 (2.7)	9 (2.7)	2.6 (3)	< 0.001	CN,SCD > MCI	20/21/17
MoCA total scrore	27.3 (2.1)	25.9 (2.2)	21.3 (3.5)	< 0.001	CN,SCD > MCI	22/21/17
CDR - sum of boxes	0.1 (0.4)	0.2 (0.5)	1.8 (1.3)	< 0.001	CN,SCD < MCI	22/21/18
CCI - self (12 items)	15.4 (2.3)	26.6 (5.5)	35.2 (10.6)	< 0.001	CN < SCD < MCI	21/21/17

Abbreviations: A*β*, amyloid-*β*; APOE, apolipoprotein E; RAVLT, Rey Auditory Verbal Learning Test; MoCA, Montreal Cognitive Assessment; CDR, Clinical Dementia Rating scale; CCI, Cognitive Change Index; CN, cognitively normal; MCI, mild cognitive impairment; SCD, subjective cognitive decline.

† Tau positivity: (+) = SUVR ≥ 1.23; SUVR = standard uptake value ratio.

‡ Amyloid positivity: (+) = [18F]Florbetapir SUVR ≥ 1.1 or [18F]Florbetaben SUVR ≥ 1.2.

**Table 2:** A list of highly significant pathways (i.e., valid connection) with *P* values less than 0.01.

Hemisphere	TAU_up_ ROI	TAU_down_ ROI	*p* ^1^	*p* ^2^
L	middle temporal	inferior parietal	0.00014	0.00007
L	middle temporal	STS	0.00012	0.00006
L	inferior temporal	lateral occipital	0.00025	0.00005
L	inferior temporal	inferior parietal	0.00003	0.00006
L	fusiform	lingual	0.00019	0.00019
L	fusiform	lateral occipital	0.00160	0.00025
L	lateral occipital	superior parietal	0.00016	0.00150
L	lateral occipital	pericalcarine	0.00170	0.00750
L	lateral occipital	cuneus	0.00076	0.00048
L	inferior parietal	supramarginal	0.00450	0.00370
L	inferior parietal	superior parietal	0.00240	0.00730
L	STS	superior temporal	0.00490	0.00270
L	precuneus	isthmus cingulate	0.00120	0.00091
L	postcentral	paracentral	0.00580	0.00720
R	middle temporal	inferior parietal	0.00036	0.00090
R	middle temporal	STS	0.00230	0.00150
R	inferior temporal	lateral occipital	0.00002	0.00030
R	inferior temporal	inferior parietal	0.00038	0.00100
R	fusiform	lingual	0.00009	0.00034
R	fusiform	lateral occipital	0.00014	0.00040
R	lateral occipital	superior parietal	0.00170	0.00610
R	lateral occipital	cuneus	0.00024	0.00002
R	superior parietal	precuneus	0.00460	0.00930
R	superior parietal	postcentral	0.00390	0.00044
R	precuneus	isthmus cingulate	0.00043	0.00040

*P*^1^ denotes the *P* value for the association between TAU_up_ and Diff_WM_ (i.e., tau in the seed ROI and the diffusion metric along its connecting WM tract) in that particular connection. In the same connection, *P*^2^ denotes the *P* value for the association between Diff_WM_ and TAU_down_ (i.e., the diffusion metric along the WM tract and tau in the tract’s destination GM ROI). *P* values less than 0.001 are further highlighted in blue. L denotes the left hemisphere and R denotes the right hemisphere.

STS: banks of the superior temporal sulcus

## Abbreviations:

AD: Alzheimer’s disease
APOE: apolipoprotein E
CCI: cognitive change index
CDR: clinical dementia rating scale
CN: cognitively normal
Da: axial diffusivity
Dr: radial diffusivity
DTI: diffusion tensor imaging
FA: fractional anisotropy
FSL: FMRIB software library
HYDI: hybrid diffusion imaging
ICVF: intra-axonal volume fraction
MCI: mild cognitive impairment
MD: mean diffusivity
MoCA: montreal cognitive assessment
MRI: magnetic resonance imaging
NODDI: neurite orientation dispersion and density imaging
OD: orientation dispersion
RAVLT: Rey auditory verbal learning test
ROI: region of interest
SCD: subjective cognitive decline
SUVR: standardized uptake value ratio
