# Supplementary material for: Tau-related white-matter alterations along spatially selective pathways
Source: Neuroimage. Author manuscript; Available in PMC 2021 Aug 14. (PMC8364310; doi:10.1016/j.neuroimage.2020.117560)
Supplement: Supplemental Material [file NIHMS1730614-supplement-Supplemental_Material.docx]

**Supplementary Table S1.** Abbreviation of diffusion metrics and their microstructural implications.

| **Model** | **Diffusion metrics** | | **Unit** | **Intensity range** | **Microstructural implications and comments** |
| --- | --- | --- | --- | --- | --- |
| DTI | FA | AU | | 0 - 1 | Fractional anisotropy describing WM coherence; influenced by both axial diffusivity (Da) and radial diffusivity (Dr). |
|  | MD | | 10^-6^ mm^2^/s | 0 - 3000 | Mean diffusivity describing averaged diffusivity across diffusion compartments within an imaging voxel. |
|  | Da | | 10^-6^ mm^2^/s | 0 - 3000 | Axial diffusivity; influenced by axonal damage, cell infiltrations, and CSF contaminations {Kim, 2006 #244}. |
|  | Dr | | 10^-6^ mm^2^/s | 0 - 3000 | Radial diffusivity; influenced by myelination, inflammation, and CSF contamination {Song, 2005 #76}. |
| NODDI | ICVF | | AU | 0 - 1 | Volume fraction of intracellular compartment; a proxy of axonal density {Zhang, 2012 #1174;Wu, 2018 #2406; Wen et al., 2019}. |
|  | ODI | | AU | 0 - 1 | WM fiber dispersion computed from a Watson distribution {Zhang, 2012 #1174;Kodiweera, 2016 #1899}. |

**Supplementary Fig. S1.** The effect of thresholding in the backbone connection on the DDIS results.  Left: histograms of the number-of-streamline from all connections.  The connections or edges whose number of streamlines above the cut-off lines formed the backbone structure (gray sticks in the middle figures) Middle: backbone structure. Right: corresponding identified pathways. (A) top row: Backbone connection with a 10% cut-off. (B) middle row: Backbone connection with a 5% cut-off. Although more edges were included in the DDIS analyses with a 5% cut-off, the identified pathways were similar to (A). (C) bottom row: Association patterns corresponding to different binarizing thresholds from 4 % to 11% with an increment of 1%. The threshold value is listed on top of each DDIS association pattern. Overall, different thresholds created similar association patterns, but with different iteration stages. With a lower threshold, the DDIS terminated at an early iteration stage (1-red, 2-yellow, 3-green, 4-purple), whereas DDIS with a higher binarizing threshold terminated at a later stage (i.e., up to 5-brown). This demonstrated that the results are insensitive to the cut-off thresholds from 4% to 11%. A 10% cut-off was used in this study.

**
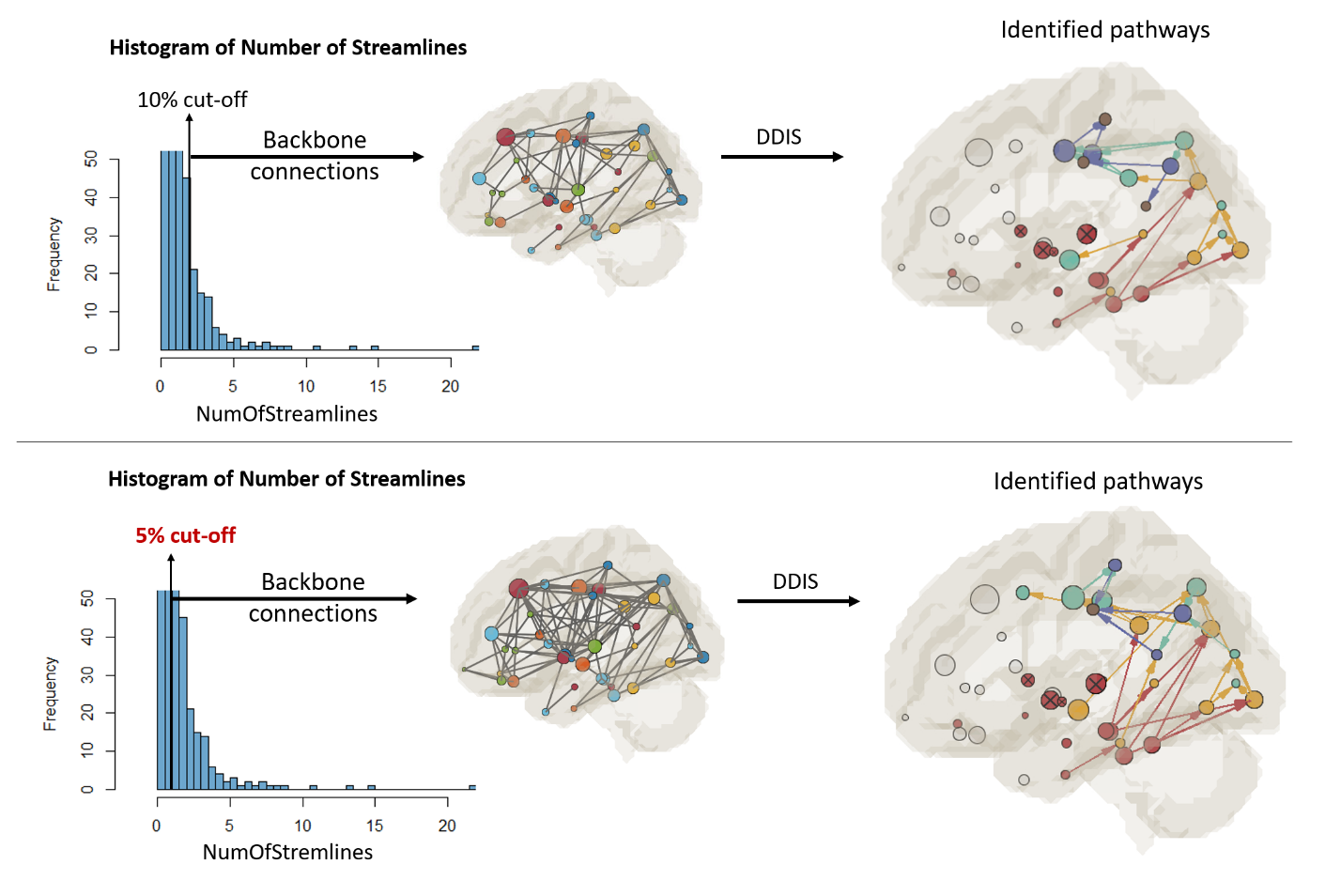
**

(A)

(B)

**
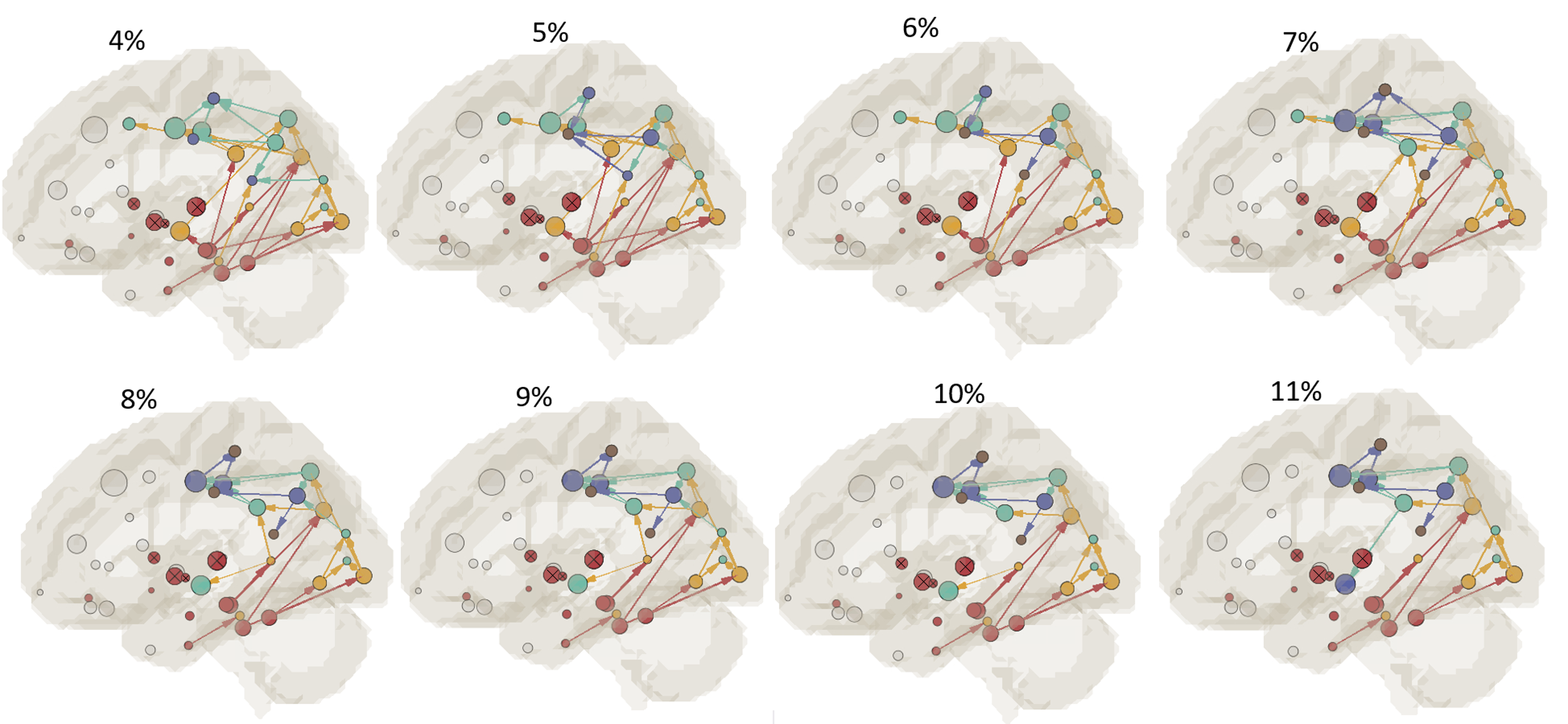
**

(C)

**Supplementary Fig. S2.** Tau+ and Amyloid+ prevalence by groups. Prevalence is defined as the percentage of subjects having tau+ in a given region-of-interest (ROI). Within-group prevalence is color coded with grey and all subject prevalence is in warm colors. CN: cognitively normal; SCD: subjective cognitive decline; MCI: mild cognitive impairment.

**
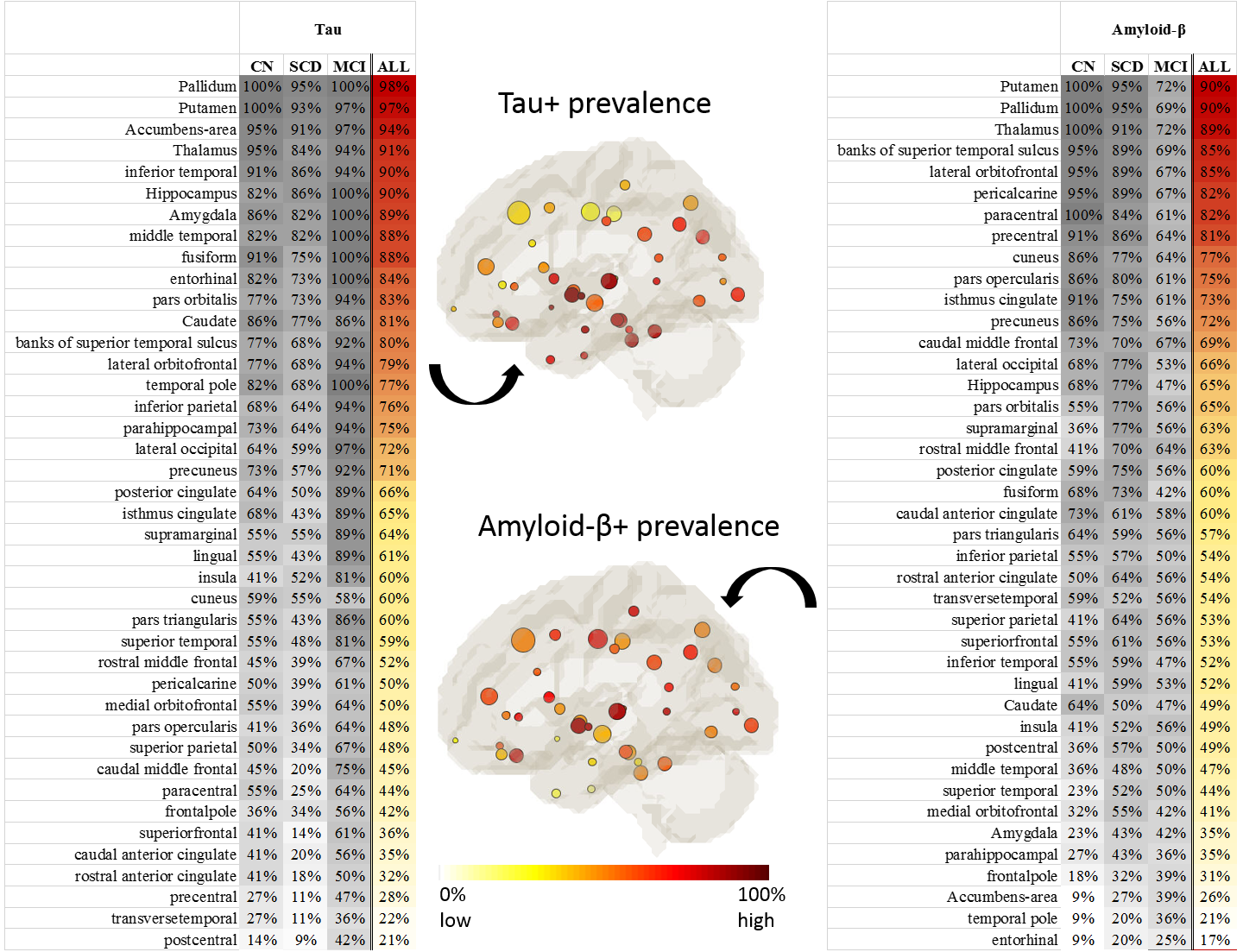
**

**Supplementary Fig. S3.** The rationale for choosing tau+ prevalence > 80% as the cut-off for the seed selection. With too few seeds (a very high cut-off), seeds may be dominant by ROIs with non-specific binding and the search will terminate after the 1st iteration. With too many seeds (a very low cut-off), within seeds connections will be overlooked as the searching process does not include seed-to-seed connections to avoid circulation. To assess the effect of the seed selection threshed on the results, the DDIS association patterns for different numbers of seeds (red nodes, ranging from 5 to 17) are illustrated above. The off-target tau binding ROIs are denoted with a cross inside the circle and include pallidum (1^st^ ROI in the tau+ prevalence, Fig. 2 or Fig. S2), putamen (2^nd^), thalamus (4^th^), and caudate (12^th^). With different numbers of the initial seeds, the nodes and edges may be identified at different stages of the iteration, labeled by different colors. Overall, the DDIS association patterns were relatively stable and insensitive to the seed selection thresholds. The 80% cut-off with 14 see ROIs (1/3 of all ROIs) was used in this study.

**
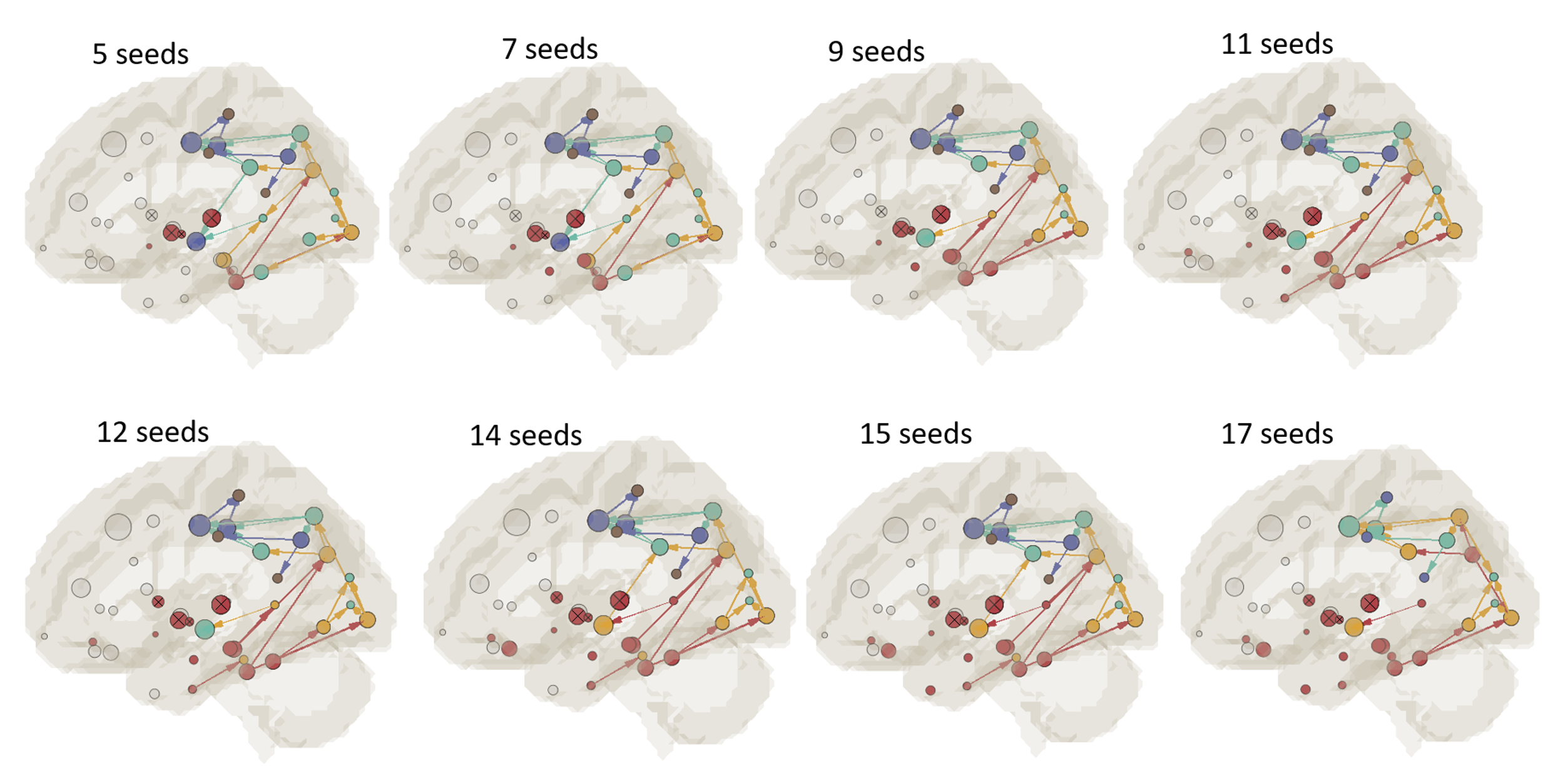
**

**Supplementary Fig. S4**. Percentage of connections identified in bootstrap samples. This shows the level of confidence for each established pathway in the main pattern (Fig. 5). The number on each connection indicates the percentage that this connection was identified as an association pathway in bootstrap samples. The early connections were more robust as the majority of these connections were identified in the bootstrap samples, while later connections were more varied as they were identified in fewer bootstrap samples.

**
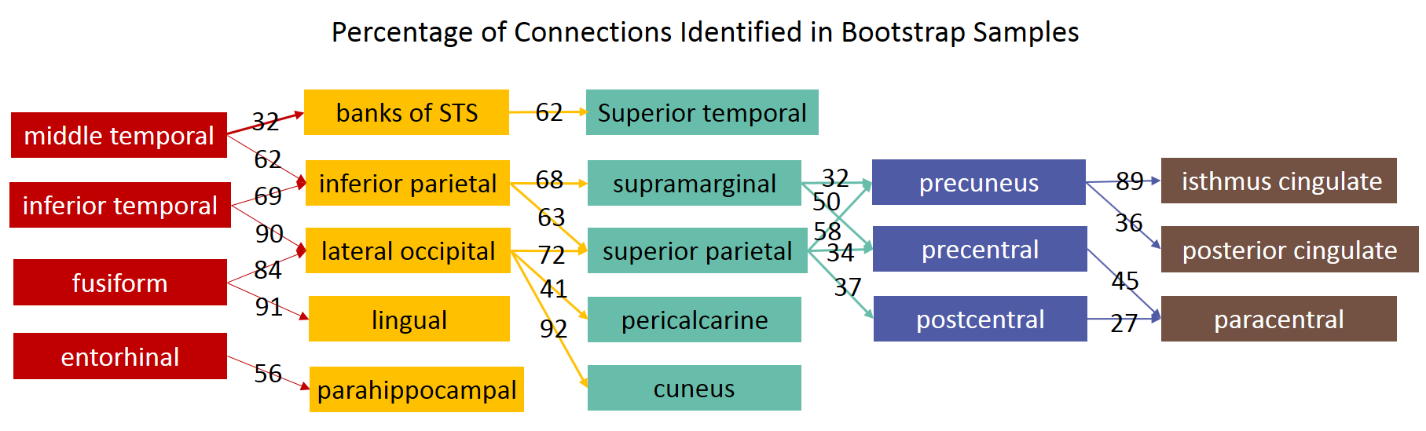
**

**Supplementary Fig. S5.** Summary statistics of associations between amyloid-β and the diffusion metrics using DDIS framework. “# pathways” denotes the total number of detected association pathways, each of which contains a dual association between amyloid-β in both ends and a diffusion metric in the connection. *r^2^_diff_* describes additional variance explained by a diffusion metric in the multivariate regression model that controls for age and sex. “Direction of change” denotes the direction of change in the diffusion metric when amyloid-β increases in the gray-matter ROIs.

**
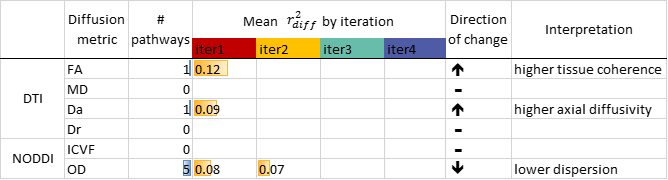
**
